# American Pharmaceutical Association

**Published:** 1878-11

**Authors:** 


### AMERICAN PHARMACEUTICAL ASSOCIATION.

The meeting of this Association for the year 1877 was held in Toronto, Canada, and adjourned to meet in Atlanta on the 2nd of September, 1878. Owing to the yellow fever scourge in the South-west, however, the meeting at that time was postponed to the 26th of November of the present year, at which time it will be held in this city.

A good attendance is expected, and Atlanta druggists and the citizens generally will welcome visitors on the occasion with their usual efforts to make comfortable and happy strangers in our midst. We hope physicians of the city and surrounding country will feel an interest in the deliberations of this useful association.

